# Global burden of mental disorders among children aged 5–14 years

**DOI:** 10.1186/s13034-018-0225-4

**Published:** 2018-04-12

**Authors:** Marie Laure Baranne, Bruno Falissard

**Affiliations:** CESP, INSERM U1018, Université Paris-Saclay, Université Paris-Sud, UVSQ, APHP, Paris, France

**Keywords:** Mental health, Children, Burden of disease

## Abstract

**Background:**

The global burden of disease (GBD) study provides information about fatal and non-fatal health outcomes around the world.

**Methods:**

The objective of this work is to describe the burden of mental disorders among children aged 5–14 years in each of the six regions of the World Health Organisation. Data come from the GBD 2015 study. Outcomes: disability-adjusted life-years (DALYs) are the main indicator of GBD studies and are built from years of life lost (YLLs) and years of life lived with disability (YLDs).

**Results:**

Mental disorders are among the leading causes of YLDs and of DALYs in Europe and the Americas. Because of the importance of infectious diseases, mental disorders appear marginal in Africa for YLLs although they play an important role in YLDs there. Because the epidemiological transition that has taken place in Europe and the Americas (i.e., a switch from acute and infectious conditions to chronic and mental health issues) is likely to happen sooner or later across the entire planet, mental health problems in youth are likely to become one of the main public health challenges of the twenty-first century.

**Conclusion:**

These results should improve health care if policy-makers use them to develop health policies to meet the real needs of populations (especially children) today.

**Electronic supplementary material:**

The online version of this article (10.1186/s13034-018-0225-4) contains supplementary material, which is available to authorized users.

## Background

Determination of the health problems that most often or most severely affect specific populations is necessary to optimise health services and prioritise health policies. Until recently, the indicators most frequently chosen to understand population needs were mortality, life expectancy, and their causes and risk factors. As advances in medical knowledge have increased life expectancy in most of the world’s regions, the dichotomy between fatal vs non-fatal effects has become much less relevant [1]. In 1992, the World Health Organisation (WHO) asked C. Murray and his collaborators to develop a more comprehensive indicator that would reflect not only the mortality but also the level of disability due to particular diseases. The Lancet published four articles applying this perspective in 1997, all based on the concept of the global burden of disease (GBD) [2]. They assessed GBD through a new indicator called the DALY, for disability-adjusted life years [3]. Since then, studies of DALYs and GBD, because they provide a comprehensive picture of population needs, have become essential part of the public health literature. Technically, DALYs are estimated from mortality and disability data. Disability is estimated from the prevalence of a given disease, its average duration, and a subjective appreciation of its day-to-day impact (often obtained through revealed preference surveys in the general population) [2]. The first set of GBD studies did not clearly identify psychiatric disorders, which were grouped with neurologic disorders. The situation was even worse for most child and adolescent psychiatric disorders, which have been seriously considered only since 2010 [4].

Although some papers have already presented the results of the most recent GBD studies of young people, most of their analyses were conducted at the level of the planet and thus masked the huge specificities that exist between regions [5]. For this reason, these analyses were unable to interpret the global weight of mental disorders adequately in the paediatric population.

The objective of the present paper is to describe and analyse the global burden of mental disorders among children aged 5–14 years throughout the world from the latest data available (GBD 2015), with a focus on each WHO region and an explicit summary of the relative importance of mortality and disability in each.

## Methods

For GBD 2015, diseases were defined according to the International Classification of Diseases, 10th revision (ICD-10), and organised in a hierarchical classification [6, 7]. The first level of this classification comprises three main disease groups: communicable diseases (group 1), non-communicable diseases (group 2), and injuries (group 3). These three groups are divided in 21 categories. The communicable disease categories include, for example, infectious and parasitic diseases and neonatal conditions. Categories of non-communicable diseases include mental disorders, as well as malignant neoplasms and endocrine, blood, and immune disorders. Injuries regroup intentional and non-intentional injuries. The third level of classification is closer to the usual ICD-10 categories. Mental disorders, for instance, are divided into 13 subcategories: major depressive disorders, dysthymia, bipolar disorders, schizophrenia, alcohol use disorders, drug use disorders, anxiety disorders, eating disorders, autism spectrum disorders (reported in the GBD database as Autism and Asperger syndrome), conduct disorders, attention-deficit/hyperactivity disorder (ADHD), idiopathic intellectual disability, and other mental and behavioural disorders.

The aggregate data from the 2015 GBD study are freely available [7]. This dataset provides DALYs for the six WHO regions—Africa (AFR), the Americas region (AMR), South-East Asia (SEAR), Europe (EUR), the Eastern Mediterranean region (EMR), and the West Pacific region (WPR)—for each sex and for seven age groups: < 28 days, 1–59 months, 5–14, 15–29, 30–49, 50–69 years, and 70 and older [6].

Formally, DALYs are the sum of years of life lost (YLLs) and years lost due to disability (YLDs) for disorder (d), age (a), sex (s), and year (t).

$$ {\text{DALY }}\left( {{\text{d}},{\text{ a}},{\text{ s}},{\text{ t}}} \right) \, = {\text{ YLL }}\left( {{\text{d}},{\text{ a}},{\text{ s}},{\text{ t}}} \right) \, + {\text{ YLD }}\left( {{\text{d}},{\text{ a}},{\text{ s}},{\text{ t}}} \right). $$

YLLs and YLDs are estimated as follows:

$$ {\text{YLL}}\, = \,{\text{N}}\left( {{\text{d}},\,{\text{s}},\,{\text{a}},\,{\text{t}}} \right)\, \times \,{\text{L}}\left( {{\text{s}},\,{\text{a}}} \right), $$

where: N(d, s, a, t) is the number of deaths due to disorder (d) for a given age (a) and sex (s) in year (t). L(s, a) is a function specifying the number of YLLs for a person of sex (s) dying at age (a).

The equation for YLDs is:

$$ {\text{YLD}}\, = \,{\text{P}}\left( {{\text{d}},\,{\text{s}},\,{\text{a}},\,{\text{t}}} \right)\, \times \,{\text{DW}}\left( {{\text{d}},\,{\text{s}},\,{\text{a}}} \right)\, \times \,{\text{L}}\left( {{\text{d}},\,{\text{s}},\,{\text{a}},\,{\text{t}}} \right), $$

where: P(d, s, a, t) = prevalence of the disorder of interest (d) at age (a) and sex (s); DW(d, s, a) = disability weight for the disorder of interest (d) at age (a) and sex (s); L(d, s, a, t) = average duration of the case until remission or death (years).

In the GBD 2015 study, disability weights were obtained from two international surveys in the general populations of nine countries in 2011 and 2013: Bangladesh, Indonesia, Peru, Tanzania, the USA, Hungary, Italy, the Netherlands and Sweden [8, 9]. Two approaches were used: face-to face interviews and online surveys. The method relied on the revealed preference paradigm. More precisely, respondents were asked to determine the healthier of two situations in a series of questions. Concerning ADHD, for example, one question was:

“Who do you think is healthier overall (in terms of having fewer physical or mental limitations on what the person can do in life), the first or the second person:”

- Person 1: “ADHD: the person is hyperactive and has difficulty concentrating, remembering things, and completing tasks.”
- Person 2: “Partially controlled asthma: The person has wheezing and coughing once a week, which causes some difficulty with daily activities.”

The duration of each disability L(d, s, a, t) until remission or death was estimated by experts on the basis of a literature review.

The GBD 2015 study differed in some aspects from the previous studies [7]:

- Age weighting is now uniform across the lifespan; earlier versions had assigned less weight to years of healthy life lost at extreme ages [10].
- YLDs are now based on prevalence estimates, although the earlier GBD studies used disease incidence preferentially.
- YLDs are now adjusted for independent comorbidities.
- Disease weights and prevalence estimation have been revised and updated.

At classification level two, where mental disorders appear as a broad category, we extracted the five main categories of disorders causing loss of DALYs in each of the six WHO regions, for children and adolescents aged from 5 to 14 years of each sex. At level 3 (the level of specific disorders), we considered the 20 disorders that explain the greatest losses of DALYs.

Then we standardised DALYs by the size of the population aged 5–14 years:

DALY1000 = Number of DALYs/Total relevant (youth) population in the region.

We also estimated the relative trends of the DALY1000 between 2000 and 2015 in each region.

$$ \left[ {\left( {{\text{DALY1}}000{\text{ in 2}}0 1 5\,{-}\,{\text{DALY1}}000{\text{ in 2}}000} \right)/\left( {{\text{DALY1}}000{\text{ in 2}}000} \right)} \right]\,*\, 100 $$

To evaluate the relative weight of death and disability in the burden of mental disorders, we explored the relative proportion of YLLs and YLDs. We also compared YLDs caused by mental disorders with all causes of YLDs in group 2 (non-communicable diseases).

## Results

**Fig. 1:**
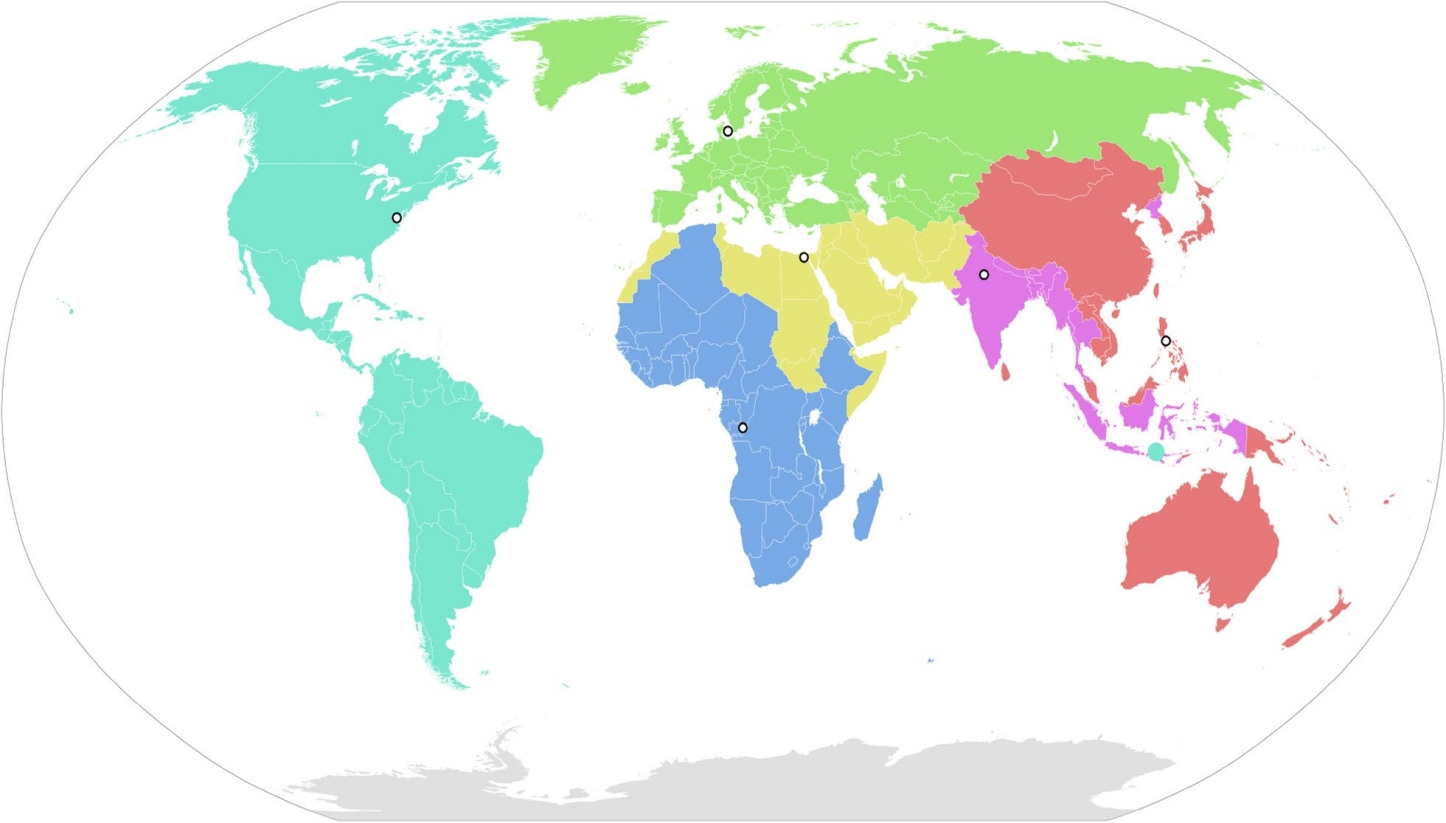
Map of the borders of the WHO regions considered in the GBD 2015 study

The WHO regions are presented in Fig. 1.

### Burden of mental disorders in 2000 and in 2015 in children aged 5–14 years

**Fig. 2:**
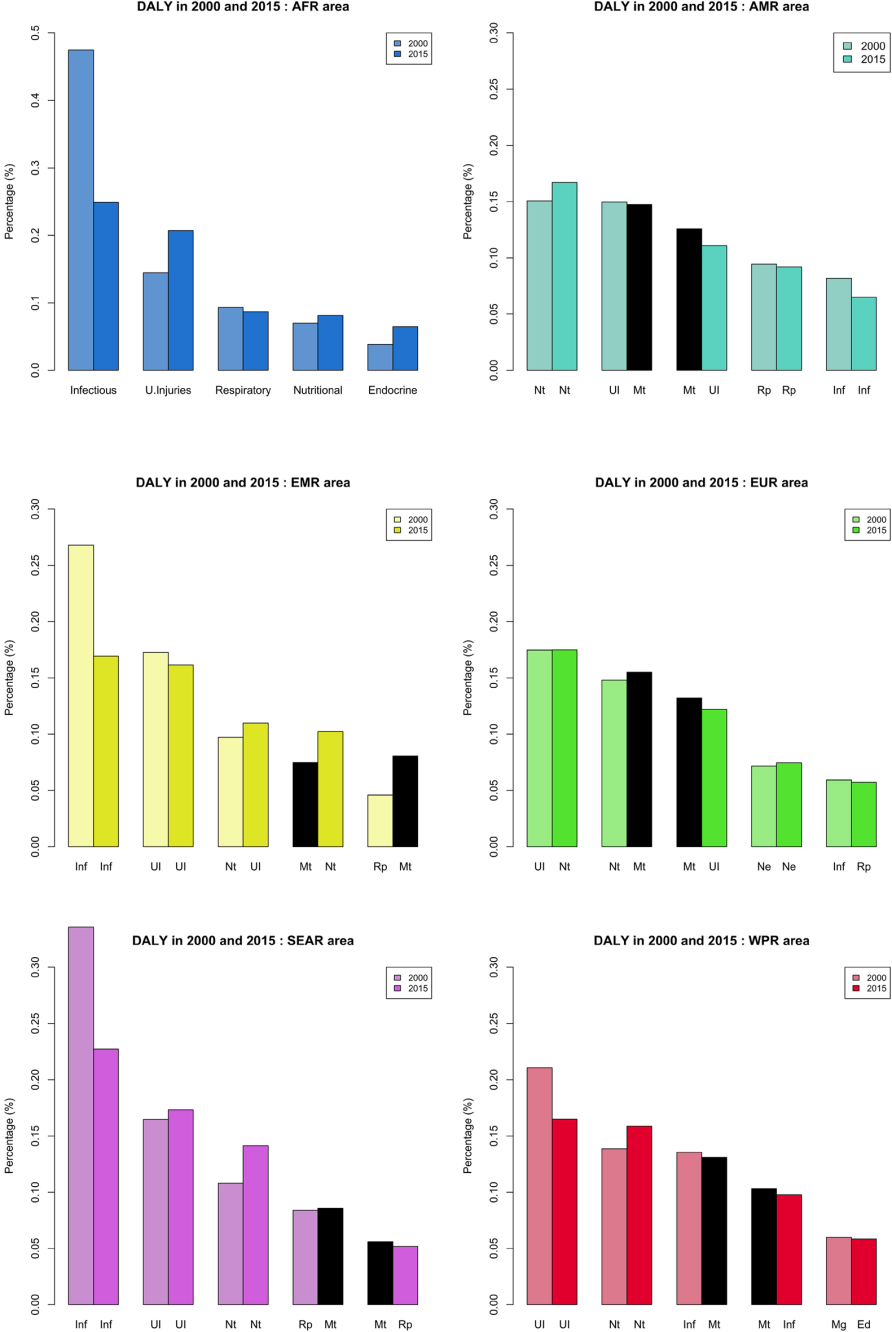
The five leading level-2 causes of DALY1000 in each WHO region for the 5–14-year age group in 2000 and in 2015. *Nt* nutritional disorders, *UI* unintentional injuries, *Mt* mental and substance use disorders, *Rp* respiratory diseases, *Inf* infectious and parasitic diseases, *Nt* nutritional disease, *Ne* neurologic disorders, *Sk* skin diseases, *Ed* endocrine, blood, immune disorders, *Mg* malignant neoplasms

**Fig. 3:**
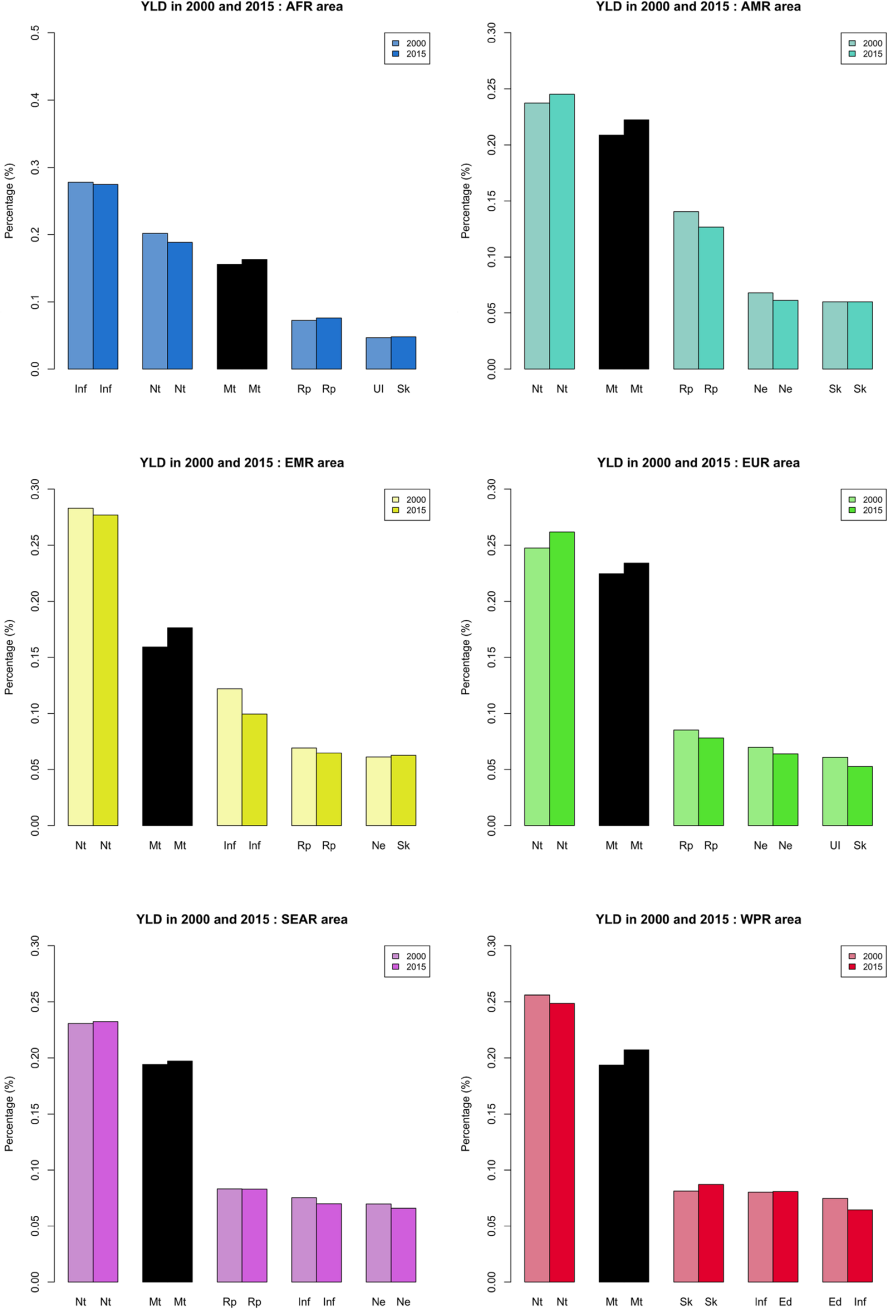
The five leading level-2 causes of YLDs per 1000 inhabitants in each WHO region for the 5–14-year age group in 2000 and 2015. *Nt* nutritional disorders, *UI* unintentional injuries, *Mt* mental and substance use disorders, *Rp* respiratory diseases, *Inf* infectious and parasitic diseases, *Nt* nutritional disease, *Ne* neurologic disorders, *Sk* skin diseases, *Ed* endocrine, blood, immune disorders, *Mg* malignant neoplasms

In the Americas and Europe, mental disorders in 2000 ranked third among the causes of DALYs (Fig. 2). By 2015, they had reached second place (Fig. 3).

In 2000, mental disorders were the fourth leading cause of DALYs in South-East Asia, the Eastern Mediterranean, and the Western Pacific. In 2015, their rank remained stable in South-East Asia, fell to fifth place in the Eastern Mediterranean (likely because of wars in this region, which increased the number of deaths due to injuries), and reached third place in the Western Pacific.

In Africa, mental disorders were not in the top five causes of losses of DALYs in either 2000 or 2015. Infectious diseases were the most prevalent cause of DALYs among children of this region. In Europe, from 2000 to 2015, the impact of infectious diseases on DALYs decreased, while that of mental disorders increased. The same was true in the Western Pacific and South-East Asia and to a lesser extent in the Americas. Only in the Eastern Mediterranean did this situation differ.

In addition to this period effect, there is also an income effect: the continent with the highest gross domestic product has globally fewer problems with infectious diseases and more problems with mental disorders. This is especially true in Western Europe, where infectious diseases are no longer in the top 5 DALY causes, but mental disorders are in the first place. This phenomenon is called an epidemiological transition, which we will consider in more detail in the discussion. The epidemiologic transition concerns all age groups of a population. Our results show clearly that it is especially relevant in the 5–14 age group.

Mental disorders have an important impact on the YLDs in Africa (Fig. 3). This point is important because the organisation of health care systems depends strongly on the profile of patients with important disabilities.

### Trends of the normalised burden of mental disorders from 2000 to 2015

Because the burden of disease evaluated from DALYs depends on the population size, it is also interesting to look at a standardised estimate of the DALY, the DALY1000 described in the methods section. The course of this indicator was stable in most regions during the study period. As Table 1 shows, two regions had a relative variation in the DALY1000 between 2000 and 2015 that reached 5%: Europe and the Americas. Surprisingly, these variations moved in opposite directions. In Europe, the DALY1000 associated with mental disorders decreased by 5.3%. The simultaneous increase in the relative weight of mental disorders implies that the global health of children and adolescents aged from 5 to 14 improved substantially. In the Americas, the DALY1000 associated with mental disorders increased by 4%.

**Table 1:** Trends over time of DALYs caused by mental disorders from 2000 to 2015 in children aged 5–14 years

Region	Age (years)	Population (thousands) 2015	Population (thousands) 2000	DALY (000 s) in 2000	DALY (000 s) in 2015	DALY change 2000–2015 (%)	DALY1000 in 2015	DALY1000 in 2000	DALY1000 change 2000–2015 (%)
AFR	5–14	259,866.6	179,060.3	1,863,516	2,676,614	43.6	10.30	10.41	− 1.0
AMR	5–14	153,998.0	155,225.3	1,664,443	1,717,707	3.2	11.15	10.72	4.0
SEAR	5–14	364,810.6	348,134.3	3,429,175	3,689,155	7.6	10.11	9.85	2.7
EUR	5–14	105,064.9	120,968.3	1,378,652	1,133,424	− 17.8	10.79	11.40	− 5.3
EMR	5–14	134,825.1	120,066.3	1,404,740	1,553,569	10.6	11.52	11.70	− 1.5
WPR	5–14	221,655.7	312,317.4	2,967,064	2,139,505	− 27.9	9.65	9.50	1.6

### Principal mental disorders (level 3 of analysis) that affect losses of DALYs (Additional file 1: Table S1 and Additional file 2: Table S2)

We focus here on the 20 diseases (considered fairly broadly) that caused the most DALYs. Except for Africa (which has no mental disorder in its top 20), most regions typically had four mental disorders in the top 20 in both 2000 and 2015: conduct disorders and anxiety disorders were respectively first and second among mental disorders, while major depressive disorders and autism-Asperger syndrome alternated between ranking third or fourth, depending on continent and period, although one or the other was occasionally surpassed by idiopathic intellectual disability.

Among boys, the most common mental disorders in the top 20 causes of losses of DALYs in 2015 were conduct disorders (in all regions), autism-Asperger syndrome, and anxiety disorders. Among girls they were anxiety disorders, conduct disorders, and major depressive disorder.

### Relative importance of YLLs and YLDs

As expected, in the 5–14-year age group, the importance of YLLs due to mental disorders was marginal. YLDs are clearly the main component of these DALYs.

## Discussion

Without data about DALYS, YLLs, and YLDs, it is difficult to determine health priorities rationally. When people hear the words leukaemia, stroke, Alzheimer, or schizophrenia, they experience feelings, emotions, and often compassion, but according to a metric that is not necessarily fair. For example, most consider that Hodgkin disease is clearly much more severe than anorexia nervosa, although the prognosis for survival is the same for both [11, 12]. Mental disorders, which are still considered mysterious in most societies because they are supposed to affect the mind more than the body, are often neglected and even denied by populations. Policy-makers are thus often tempted to cut spending in this domain. This is even truer for child and adolescent psychiatry, where some pathologies, such as conduct disorders, are considered deviance rather than a health problem that requires compassion and care. For a long time, public health professionals did not significantly help repair this injustice; their quantitative work was limited to mortality statistics, while many important child and adolescent psychiatric disorders, such as autistic spectrum disorder or anxiety disorders, have only a marginal impact on mortality, although their effect on daily life can be extreme.

The development and recording of DALYs and YLDs are thus an important breakthrough in the context of global health. We now have data covering several years for DALYs and YLDs due to mental disorders for 5–14-year-olds in the different regions of the globe. This paper focuses on this crucial statistic.

The impact of mental disorders on the burden of disease among children aged 5–14 years appears to be very strong in the Americas and in Europe. In other regions, mental disorders also play a notable role that will surely increase in the future, as they undergo the “epidemiological transition”. Omran describes this concept as “focus[ing] on the complex change in patterns of health and disease and on the interactions between these patterns and their demographic, economic and sociologic determinants and consequences” [13]. Europe and the Americas are the two regions where the epidemiological transition was first observed, because of their high level of development. This transition begins with a decrease in mortality from infectious and epidemic diseases and then the modification of the health problems encountered by populations, which because they are living longer, face new and different health challenges [13]. An epidemiological transition results in the regression of communicable diseases and injuries, at the same time as non-communicable diseases, such as mental disorders but also degenerative disorders and cancers, tend to grow in importance. These changes have already taken place in Europe and the Americas. This trend is also notable but less advanced in the Western Pacific and South-East Asia regions. In the Eastern Mediterranean, the rank of mental disorders among 5–14 years has declined over the study period, while intentional injuries are now in the top five. This finding is most likely due to recent war in this region. This hypothesis is buttressed by the major increase between 2000 and 2015 of “Collective violence and legal intervention” as a cause of DALYs. In Africa, where infectious diseases remain common and lethal, mental disorders appear to play a lesser role. Nevertheless, the level 3 classification of diseases, which is more precise, showed the appearance of conduct disorders among 5–14 year-old boys in 2015. This may reflect the first sign of an epidemiological transition.

When we focus on the YLDs, which give an overview of the morbid impact of diseases, we see that even in emerging regions, the burden of mental disorders is already high and constant over time.

Furthermore, the many comorbidities make it difficult to disentangle the specific role of disease categories. For example, unintentional injuries play an important role in disability in many regions, but several mental disorders, such as conduct disorders and attention-deficit/hyperactivity disorders, are associated with higher rates of injuries [4]. This highlights the difficulties in assessing the actual prevalence of mental disorders in less developed areas. Populations with less access to health and psychiatric care are diagnosed less often, and the consequences of their disorders are not considered to be health-related. In 2011, Gore et al. analysed the burden of disease in the 10–24 age group from earlier GBD date and showed the predominant place of mental disorders [14]. Even though these diseases were aggregated with neurologic disorders, the authors were able to show the importance of specific psychiatric disorders and discussed the difficulties in assessing them, as well as their low priority for researchers, especially in low- and middle-income countries.

This work obviously has some limitations. The calculation of DALYs requires the estimation of many parameters that are known only approximately, at best. Mortality statistics are likely to be accurate because most countries have a system for registering deaths [15]. DALYs are assessed from three parameters (prevalence, disability weight, and average duration of the case until remission or death) evaluated from multiple points of view (epidemiological surveys, opinion surveys, and expert knowledge). These multiple sources of information present many possible sources of error that magnify the uncertainty of the DALY estimates [16]. The individualisation of child psychiatric disorders is a breakthrough but also a challenge. Estimating its prevalence is difficult; it requires, among other things, a good health care system.

Furthermore, disease weighting is likely to be the trickiest part of these estimations. Respondents had to choose between two heath situations explained in simplistic terms. For example, the description of ADHD does not come close to an accurate description of the reality experienced by patients and their families in their daily lives: “is hyperactive and has difficulty concentrating, remembering things, and completing tasks” [7]. This presentation ignores the impact of ADHD on social exclusion, stigmatisation, school difficulties, accidents, etc. The consequence is a quite debatable relative ranking [17]. For example, ADHD has a disease weight of 0.045, whereas symptomatic benign prostatic hypertrophy (the lay description of which is: “feels the urge to urinate frequently, but when passing urine, it comes out slowly and is sometimes painful”) has a disease weight of 0.067. Disease weighting is a powerful approach toward capturing the population’s point of view of health situations. It is limited, however, by the fact that it collects abstract and subjective representations of people who have never dealt with or perhaps even seen the disorder, as opposed to the real experience of patients and their families. Another important limitation comes from the definition of burden of disease, which does not include the impact of disorders on caregivers [18, 19]. In many ways, mental disorders cannot be considered standard diseases, especially among children. Psychiatric diseases in a population aged 5–14 years must be considered systemic. That is, they affect a community, most often a family, which must be considered as a whole [20].

This paper is intended as an urgent signal of alarm to national and international public health institutions and policy-makers. The world is experiencing an epidemiologic transition. The relative burden of disease of mental disorders among children aged 5–14 years is increasing and will accelerate even more in the near future. This change and the problems accompanying it will require specific responses. Psychiatric diseases in children must be considered specifically. Planning a vaccination program or an antibiotic prescription is very different from organising a global policy for mental health and psychiatric care. It requires a long-term perspective, specially trained professionals, and careful preparation in view of the numerous obstacles that must be anticipated, including local representations of psychiatric diseases, the time necessary for effectiveness, and the extensive resources, financial and human, that will be required. This is a major challenge.

## Conclusions

The recognition of mental disorders in children and their consequences has improved, thanks, in particular, to very macroscopic studies such as the Global burden of disease study. Our description shows two major trends: the rates of mental disorders per inhabitant have remained stable over time and at the same time the epidemiological transition has placed them among the main causes of disease burden in this age group. These results ought to lead to improved health care if policy-makers use them to develop health policies to meet the real needs of populations today. Prevention, diagnosis, treatment and family support should be organised according to these findings.

## Additional files

**Additional file 1: Table S1.** Top 20 causes of DALYs in 2000 by WHO regions, in the 5–14-year age group. Mental disorders are highlighted.

**Additional file 2: Table S2.** Top 20 causes of DALYs in 2015 by WHO regions, in the 5–14-year age group. Mental disorders are highlighted.

## Abbreviations

ADHD: attention deficit and hyperactivity disorders
AFR: African region
AMR: Americas
DALY: disability-adjusted life-years
EMR: Eastern Mediterranean region
EUR: European region
GBD: Global burden of disease
SEAR: South East Asian region
WHO: World Health Organisation
WPR: West Pacific region
YLDs: years of life lived with disability
YLDs: years of life lost
