# Supplementary material for: Global burden of mental disorders among children aged 5–14 years
Source: Child Adolesc Psychiatry Ment Health. 2018 Apr 12;12:19. doi: 10.1186/s13034-018-0225-4 (PMC5896103; doi:10.1186/s13034-018-0225-4)
Supplement: Supplementary file 1 — Additional file 1: Table S1. Top 20 causes of DALYs in 2000 by WHO regions, in the 5–14-year age group. Mental disorders are highlighted. [file 13034_2018_225_MOESM1_ESM.docx]

| 2000 | | |
| --- | --- | --- |
| AFR | Measles 10.3% | Sickle cell disorders 2.98% |
|  | Diarrhoeal diseases 9.99% | Malaria 2.80% |
|  | Lower respiratory infections 8.94% | Road injury 2.66% |
|  | Meningitis 5.86% | Syphilis 2.17% |
|  | Other infectious diseases 4.35% | Asthma 1.64% |
|  | Other unintentional injuries 3.94% | Falls 1.14% |
|  | Iron deficiency anaemia 3.72% | African Trypanosomias 1.37% |
|  | Drowning 3.50% | Collective violence and legal intervention 1.36% |
|  | HIV/AIDS 3.26% | Congenital heart anomalies 1.3% |
|  | Protein energy malnutrition 3.05% | Fire, heat and hot substances 1.29% |
| AMR | Iron deficiency anaemia 14.23% | Other infectious diseases 2.22% |
|  | Asthma 8.41% | Epilepsy 1.93% |
|  | Road injury 5.07% | Interpersonal violence 1.89% |
|  | **Conduct disorder 3.82%** | Leukaemia 1.87% |
|  | Skin diseases 3.69% | **Autism and Asperger syndrome 1.78%** |
|  | Other unintentional injuries 3.58% | Diarrhoeal diseases 1.67% |
|  | **Anxiety disorders 3.3%** | **Major depressive disorder 1.64%** |
|  | Drowning 2.92% | Other congenital anomalies 1.55% |
|  | Lower respiratory infections 2.85% | Congenital heart anomalies 1.54% |
|  | Migraine 2.36% | Other neurological conditions 1.34% |
| SEAR | Iron deficiency anaemia 9.67% | Asthma 2.36% |
|  | Diarrhoeal diseases 9.47% | Meningitis 2.12% |
|  | Lower respiratory infections 7.56% | Skin diseases 2.05% |
|  | Measles 4.56% | Falls 1.97% |
|  | Other infectious diseases 4.20% | Encephalitis 1.94% |
|  | Drowning 4.18% | **Conduct disorder 1.63%** |
|  | Other unintentional injuries 3.97% | Malaria 1.50% |
|  | Tuberculosis 3.31% | Tetanus 1.32% |
|  | Road injury 3.04% | Congenital heart anomalies 1.18% |
|  | Epilepsy 2.56% | Poisonings 1.16% |
| EUR | Iron deficiency anaemia 14.35% | Migraine 2.5% |
|  | Asthma 5.07% | Falls 2.49% |
|  | Drowning 4.17% | Congenital heart anomalies 2.04% |
|  | Road injury 4.02% | **Major depressive disorder 1.85%** |
|  | **Conduct disorder 4.01%** | Leukaemia 1.77% |
|  | Lower respiratory infections 3.79% | **Autism and Asperger syndrome 1.74%** |
|  | Other unintentional injuries 3.49% | Other infectious diseases 1.71% |
|  | **Anxiety disorders 3.36%** | Other neurological conditions 1.43% |
|  | Skin diseases 3.03% | Meningitis 1.39% |
|  | Epilepsy 2.98% | Thalassaemias 1.36% |
| EMR | Iron deficiency anaemia 8.55% | **Conduct disorder 2.24%** |
|  | Measles 5.94% | Skin diseases 2.05% |
|  | Other infectionus diseases 5.27% | Congenital heart anomalies 2.03% |
|  | Diarrhoeal diseases 4.90% | Epilepsy 1.95% |
|  | Other unintentional injuries 4.77% | Meningitis 1.89% |
|  | Road injury 4.76% | **Anxiety disorders 1.73%** |
|  | Lower respiratory infections 3.69% | Thalassaemias 1.47% |
|  | Drowning 3.64% | Collective violence and legal intervention 1.44% |
|  | Asthma 3.51% | Other circulatory diseases 1.43% |
|  | Tuberculosis 2.44% | Haemorrhagic stroke 1.42% |
| WPR | Iron deficiency anaemia 13.42% | **Anxiety disorders 2.71%** |
|  | Drowning 9.59% | Lower respiratory infections 2.44% |
|  | Road injury 6.23% | Other unintentional injuries 1.98% |
|  | Skin diseases 4.37% | Congenital heart anomalies 1.88% |
|  | Asthma 3.19% | **Autism and Asperger syndrome 1.66%** |
|  | Thalassaemias 3.11% | Epilepsy 1.61% |
|  | Leukaemia 3.04% | Other circulatory diseases 1.55 |
|  | Other infectious diseases 2.97% | Preterm birth complications 1.31% |
|  | Tuberculosis 2.8% | Falls 1.25% |
|  | **Conduct disorder 2.76%** | Diarrhoeal diseases 1.24% |

Table S1. Top 20 causes of DALYs in 2000 by WHO regions, in the 5-14-year age group. Mental disorders are highlighted.
