# Supplementary material for: Global burden of mental disorders among children aged 5–14 years
Source: Child Adolesc Psychiatry Ment Health. 2018 Apr 12;12:19. doi: 10.1186/s13034-018-0225-4 (PMC5896103; doi:10.1186/s13034-018-0225-4)
Supplement: Supplementary file 2 — Additional file 2: Table S2. Top 20 causes of DALYs in 2015 by WHO regions, in the 5–14-year age group. Mental disorders are highlighted. [file 13034_2018_225_MOESM2_ESM.docx]

| 2015 | | |
| --- | --- | --- |
| AFR | Lower respiratory infections 10.55% | Road injury 2.75% |
|  | Diarrhoeal diseases 8.05% | Protein energy malnutrition 2.37% |
|  | Meningitis 6.46% | Syphilis 2.17% |
|  | Other infectious diseases 5.70% | Asthma 2.02% |
|  | HIV/AIDS 4.68% | Congenital heart anomalies 1.95% |
|  | Malaria 4.51% | Falls 1.83% |
|  | Iron deficiency anaemia 4.48% | Measles 1.66% |
|  | Other unintentional injuries 4.24% | Exposure to mechanical forces 1.54% |
|  | Sickle cell disorders 3.88% | Fire, heat and hot substances 1.42% |
|  | Drowning 3.51% | Tuberculosis 1.41% |
| AMR | Iron deficiency anaemia 16.11% | Other infectious diseases 2.22% |
|  | Asthma 8.29% | **Major depressive disorder 2.17%** |
|  | **Conduct disorder 4.35%** | Other congenital anomalies 2.12% |
|  | Skin diseases 4.03% | Drowning 2.07% |
|  | **Anxiety disorders 3.84%** | **Autism and Asperger syndrome 2.02%** |
|  | Road injury 3.74% | Leukaemia 1.82% |
|  | Migraine 2.61% | Other neurological conditions 1.67% |
|  | Other unintentional injuries 2.51% | Congenital heart anomalies 1.67% |
|  | Lower respiratory infections 2.43% | Epilepsy 1.64% |
|  | Interpersonal violence 2.25% | Preterm birth complications 1.41% |
| SEAR | Iron deficiency anaemia 13.08% | Epilepsy 2.35% |
|  | Diarrhoeal diseases 5.17% | Tuberculosis 2.20% |
|  | Drowning 4.83% | Encephalitis 1.98% |
|  | Other infectious diseases 4.27% | Falls 1.96% |
|  | Lower respiratory infections 4.01% | Meningitis 1.71% |
|  | Other unintentional injuries 4.01% | **Anxiety disorders 1.69%** |
|  | Road injury 3.65% | Migraine 1.62% |
|  | Skin diseases 3.08% | **Idiopathic intellectual disability 1.60%** |
|  | Asthma 2.86% | **Autism and Asperger syndrome 1.35%** |
|  | **Conduct disorder 2.46%** | Acute hepatitis E 1.34% |
| EUR | Iron deficiency anaemia 17.11% | Drowning 2.55% |
|  | Asthma 5.12% | **Autism and Asperger syndrome 2.08%** |
|  | **Conduct disorder 4.62%** | Other congenital anomalies 2.07% |
|  | **Anxiety disorders 4.03%** | **Major depressive disorder 2.06%** |
|  | Skin diseases 3.52% | Other infectious diseases 2.04% |
|  | Lower respiratory infections 3.32% | Congenital heart anomalies 1.98% |
|  | Migraine 2.82% | Falls 1.93% |
|  | Road injury 2.75% | Other.endocrine blood and immune disorders 1.77% |
|  | Other unintentional injuries 2.70% | Other neurological conditions 1.67% |
|  | Epilepsy 2.67% | Thalassaemias 1.64% |
| EMR | Collective violence and legal intervention 10.03% | **Conduct disorder 2.41%** |
|  | Iron deficiency anaemia 9.24% | Skin diseases 2.26% |
|  | Other unintentional injuries 5.00% | Epilepsy 1.98% |
|  | Other infectious diseases 4.79% | Meningitis 1.93% |
|  | Lower respiratory infections 3.94% | Anxiety disorders 1.86% |
|  | Road injury 3.85% | Thalassaemias 1.61% |
|  | Diarrhoeal diseases 3.74% | Falls 1.47% |
|  | Asthma 3.62% | **Idiopathic intellectual disability 1.35%** |
|  | Drowning 3.2% | Haemorrhagic stroke 1.33% |
|  | Congenital heart anomalies 2.52% | Other congenital anomalies 1.33% |
| WPR | Iron deficiency anaemia 15.6% | Congenital heart anomalies 2.45% |
|  | Drowning 6.87% | Lower respiratory infections 2.33% |
|  | Skin diseases 5.53% | **Autism and Asperger syndrome 2.08%** |
|  | Road injury 4.67% | Other circulatory diseases 1.80% |
|  | Thalassaemias 3.99% | **Idiopathic intellectual disability 1.76%** |
|  | Asthma 3.98% | Falls 1.65% |
|  | **Conduct disorder 3.46%** | Other unintentional injuries 1.63% |
|  | Other infectious diseases 3.45% | Preterm birth complications 1.46% |
|  | **Anxiety disorders 3.40%** | Epilepsy 1.43% |
|  | Leukaemia 2.46% | Other congenital anomalies 1.39% |

Table S2. Top 20 causes of DALYs in 2015 by WHO regions, in the 5-14-year age group. Mental disorders are highlighted.
